# Practical prediction model of the clinical response to programmed death-ligand 1 inhibitors in advanced gastric cancer

**DOI:** 10.1038/s12276-021-00559-1

**Published:** 2021-02-05

**Authors:** Myung-Giun Noh, Youngmin Yoon, Gihyeon Kim, Hyun Kim, Eulgi Lee, Yeongmin Kim, Changho Park, Kyung-Hwa Lee, Hansoo Park

**Affiliations:** 1 Department of Biomedical Science and Engineering, Gwangju Institute of Science and Technology (GIST), Cheomdangwagi-ro 123, Buk-gu, Gwangju Korea; 2 Genome and Company, Pangyo-ro 253, Bundang-gu. Seoungnam-si, Gyeonggi-do Korea; 3 Department of Pathology, Chonnam National University Hwasun Hospital and Medical School, 322 Seoyang-ro, Hwasun-eup, Hwasun-gun, Jeollanam-do South Korea

**Keywords:** Predictive markers, Predictive markers, Translational research, Predictive medicine

## Abstract

The identification of predictive biomarkers or models is necessary for the selection of patients who might benefit the most from immunotherapy. Seven histological features (signet ring cell [SRC], fibrous stroma, myxoid stroma, tumor-infiltrating lymphocytes [TILs], necrosis, tertiary lymphoid follicles, and ulceration) detected in surgically resected tissues (*N* = 44) were used to train a model. The presence of SRC became an optimal decision parameter for pathology alone (AUC = 0.78). Analysis of differentially expressed genes (DEGs) for the prediction of genomic markers showed that C-X-C motif chemokine ligand 11 (*CXCL11*) was high in responders (*P* < 0.001). Immunohistochemistry (IHC) was performed to verify its potential as a biomarker. IHC revealed that the expression of CXCL11 was associated with responsiveness (*P* = 0.003). The response prediction model was trained by integrating the results of the analysis of pathological factors and RNA sequencing (RNA-seq). When trained with the C5.0 decision tree model, the categorical level of the expression of *CXCL11*, a single variable, was shown to be the best model (AUC = 0.812). The AUC of the model trained with the random forest was 0.944. Survival analysis revealed that the C5.0-trained model (log-rank *P* = 0.01 for progression-free survival [PFS]; log-rank *P* = 0.012 for overall survival [OS]) and the random forest-trained model (log-rank *P* < 0.001 for PFS; log-rank *P* = 0.001 for OS) predicted prognosis more accurately than the PD-L1 test (log-rank *P* = 0.031 for PFS; log-rank *P* = 0.107 for OS).

## Gastric cancer: Promising prediction model built on biomarker data

A prediction model that identifies patients with gastric cancer who are likely to respond well to immunotherapy has been developed by researchers in South Korea. Hansoo Park at the Gwangju Institute of Science and Technology and co-workers identified several biomarkers in gastric cancer tissues that were associated with how well patients may respond to immunotherapy treatment. They found that patients with malignant cells known as signet ring cells were least likely to respond well to immune checkpoint inhibitor drugs. Conversely, high expression levels of a gene called *CXCL11* was associated with a strong positive response to the drugs. The researchers used these and other biomarker data to build a model for selecting appropriate candidates for immunotherapy. Further research will refine this initial biomarker list for gastric cancer and help improve the model.

## Introduction

The use of immune checkpoint inhibitors (ICIs) is an emerging treatment option for a variety of solid tumors. For instance, ICIs have demonstrated good therapeutic efficacy in patients with advanced gastric cancer. To improve the efficacy of anti-programmed death-ligand 1 (anti-PD-L1) therapy in advanced gastric cancer, it is necessary to identify precise predictive biomarkers or models for the optimized selection of patients with gastric cancer who might benefit the most from immunotherapy^1^. Recent studies have suggested the tumor mutational burden as a predictive factor of survival in patients with gastric cancer following treatment with ICIs^2–5^. Microsatellite instability-high (MSI-H) and Epstein-Barr virus (EBV), for which testing is currently available in most clinical settings, have been reported as good markers for predicting the responsiveness of gastric cancer to immunotherapeutic agents^4^. Moreover, MSI-H has also been reported as an indicator of a high mutational load. Several reports have shown that an abundance of tumor-infiltrating lymphocytes (TILs), such as CD3 or CD8, is associated with a good prognosis^6–9^. Based on these findings, pembrolizumab was approved in the United States for previously treated patients with advanced gastric cancer expressing a combined positive score (CPS) ≥ 1% on the programmed death-ligand 1 (PD-L1) immunohistochemistry (IHC) test^10,11^. However, it exhibited conflicting results in numerous studies evaluating the relationship between the expression of PD-L1 and response to ICIs. Different trials with pembrolizumab or nivolumab have demonstrated a rather wide range of response rates (10–26%) in patients with metastatic gastric cancer who are administered salvage therapy without a selective biomarker or PD-L1 positivity^4,11–13^. Cottrell et al. evaluated the histological features of non–small cell lung carcinoma using anti-PD-L1-mediated “immune regression and proposed associated immune-related pathologic response” (irPR) criteria in routine hematoxylin and eosin (H&E)-stained slides and correlated them with those of the residual viable tumor^14^. Similarly, the irPR score was shown to be significant in the overall response rate and overall survival of patients with melanoma^15^. However, this approach requires a biopsy during treatment, and gastric cancer is histologically different from lung cancer and melanoma.

In this study, we trained an optimal decision model to predict the response to PD-L1 inhibitors using histological features from surgical specimens of gastric cancer and validated the accuracy of the model in biopsy tissues. In addition, we searched for differentially expressed genes (DEGs) using RNA sequencing to identify markers that could support the PD-L1 IHC test. We also performed IHC to verify the usefulness of the identified genes as biomarkers. Finally, we combined these histological characteristics and the results of genomic analysis to construct an optimal prediction model for the efficacy of PD-L1 inhibitors.

## Materials and methods

### Patient cohort and clinical data

All patients recruited for this study were referred by the research team of Professor Jeeyun Lee, Division of Hematology-Oncology, Samsung Medical Center. Enrolled patients received consultation at the Hematology-Oncology Unit of the Samsung Medical Center. Patients enrolled in this study had to meet the following criteria: (1) histologically confirmed diagnosis of gastric or gastroesophageal junction adenocarcinoma, (2) age of at least 19 y, (3) previous failure of at least one line of chemotherapy that included platinum/fluoropyrimidine, (4) willingness to undergo a procedure to obtain fresh-frozen tissue within 42 d of treatment initiation for biomarker analysis, (5) adequate organ function per protocol, (6) at least one measurable lesion according to the Response Evaluation Criteria in Solid Tumors (RECIST) v.1.124, and (7) Eastern Cooperative Oncology Group performance status of 0 or 1. All patients were naive to anti-PD-1, anti-PD-L1, or anti-PD-L2 antibodies. Clinical information was collected from the electronic medical records maintained at the electronic database of the hospital. All tumor tissues used for pathology and genomic analyses in this study were obtained anytime between day 42 and day 1 prior to the initiation of study treatment. The responsiveness to ICIs was evaluated using the Response Evaluation Criteria in Solid Tumors v1.1^16^. Patients who achieved partial response and complete response were classified as responders (R). Patients who achieved progressive disease and stable disease were classified as nonresponders (NR). This study was approved by the Institutional Review Board of Gwangju Institute Science and Technology (IRB number: 20200108-BR-50-04-02). All patients provided written informed consent before enrollment.

### Histopathology

Unstained slides of formalin-fixed and paraffin-embedded (FFPE) tissues from 100 patients with histologically confirmed gastric adenocarcinoma treated with pembrolizumab were provided by the research team of Professor Lee. We received 10 slides from a single representative section for each patient. After excluding nine patient tissues that could not be utilized due to slide damage, 44 surgically obtained large tissue specimens and 47 small tissue specimens obtained by endoscopy or needle biopsy were used to train the predictive model. H&E slides from surgical specimens (*N* = 44) and biopsied tissues (*N* = 47) for each case were independently assessed by two pathologists (M-GN and K-HL) to evaluate, select, and score the histopathological features (presence of signet ring cell (SRC) component, histologic grade, presence of fibrous stroma, presence of myxoid stroma, grade of tumor-infiltrating lymphocytes (TILs), presence of neutrophil infiltration, presence of tumor necrosis, presence of tertiary lymphoid follicles, and presence of epithelial ulceration (Fig. S1)). TILs were evaluated according to the proposed guidelines for assessment in solid tumors, as previously described by Hendry et al.^17^. The grade of TILs was evaluated as moderate-to-high if intratumoral or stromal TILs were ≥30% and as low if intratumoral or stromal TILs were <30%. The tertiary lymphoid structure was evaluated as a dense follicle of lymphocytes with germinal centers adjacent to tumor cells. All histological features except for TILs were assessed for presence only.

### RNA sequencing pipeline and analysis of differentially expressed genes

The data of all used RNA sequencing FASTQ files had already been deposited as PRJEB25780 in the European Nucleotide Archive (https://www.ebi.ac.uk/ena/data/view/PRJEB25780). The deposited data featured 45 patients, and all clinical data were provided by the research team of Professor Lee. The method of RNA sequencing has been previously described^4^. Alignment, annotation, and quality control were carried out via the RNA sequencing (RNA-seq) workflow using the bcbio-nextgen bioinformatics framework (version 1.2.4) (https://github.com/bcbio/bcbio-nextgen)^18^. In brief, raw reads were aligned to the GRCh38 (hg38) version of the human reference genome using STAR version 2.6.1d^19^. MultiQC was then used for quality control and assurance analysis of the resulting bam file by comparison to metrics gathered from bcbio-nextgen, samtools^20^, and fastqc^21^. Quantitated reads were assigned to genes (features) annotated in Ensembl and counted with featureCounts^22,23^. As recommended by the developers of the bcbio-nextgen pipeline, we loaded the gene-level counts.csv.gz and the metadata.csv.gz and used DESeq2 for gene-level analysis (https://github.com/bcbio/bcbio-nextgen/blob/master/docs/contents/bulk_rnaseq.md)^24^. Gene set enrichment analysis (GSEA) was performed using Hallmark gene set v7.2 in version 4.0.3 of GSEA software (Broad)^25,26^. The number of permutations was set to 1000, and the chosen permutation type was phenotype. Results with a *P* value and FDR less than 0.05 were considered significant. Similarly, gene set variation analysis (GSVA) was performed using Hallmark gene set v7.2 with the GSVA package in the R-4.0.2 program^27^. In the GSVA package, the “ssgsea” method was selected and analyzed with a minimum size of 10 and a maximum size of 500.

### Immunohistochemistry

We used the C-X-C motif chemokine ligand 11 (CXCL11) primary antibody (ab9955, rabbit anti-human; Abcam, Cambridge, MA, USA) at a dilution of 1:400 using an antibody diluent (GBI Labs, Bothell, WA, USA) for immunohistochemical analysis. According to the antibody datasheet, two clear cell renal cell carcinoma tissues were used as controls to validate the results. Moreover, normal gastric tissue was used as a negative control for comparison with gastric cancer tissue. After determining the staining conditions, IHC staining was performed on all nonstained slides (*N* = 91) received from the pathology test described above. All immunostained slides were individually evaluated twice by two pathologists (M-GN and K-HL) blinded to the clinical details. The cell membrane and cytoplasm of tumor cells were evaluated for the expression of CXCL11. Lymphocytes were stained to evaluate inflammatory cells infiltrating the tumor or present around the tumor. In addition, the interstitial and blood vessels around the tumor were examined. The intensity of expression was graded using a 3-tier system as strong (3+), moderate (2+), or weak (1+). The extent of expression was evaluated using a 3-tier system with cutoffs of 5 and 50%. This intensity and extent criteria were applied equally to the tumor cell membrane, tumor cell cytoplasm, TILs, and interstitial and blood vessels around tumors. Clinical information as well as PD-L1 (IHC) combined positive score (CPS) results, EBV (in situ hybridization) results, and MSI (IHC) results were received from the research team of Professor Lee. The PD-L1 (IHC) test was performed at Samsung Hospital as follows. Tissue sections were freshly cut to slices of 4 μm thickness, mounted onto Fisherbrand Superfrost Plus Microscope Slides (Thermo Fisher), and then dried at 60 °C for 1 h. IHC staining was carried out on a Dako Autostainer Link 48 system (Agilent Technologies) using a Dako PD-L1 IHC 22C3 pharmDx kit (Agilent Technologies) with an EnVision FLEX visualization system and counterstained with hematoxylin according to the manufacturer’s instructions. PD-L1 protein expression was determined using CPS as follows: the number of PD-L1-stained cells (tumor cells, lymphocytes, and macrophages) divided by the total number of viable tumor cells multiplied by 100. The specimen was considered to have PD-L1 expression if CPS ≥ 1. The MSI status was determined at the Samsung Medical Center by IHC for both MLH1 (antibody: ES05 clone; 1:100 dilution; Novocastra) and MSH2 (clone G219-1129; 1:500 dilution; Cell Marque) in FFPE tissue sections and polymerase chain reaction (PCR) analysis of 5 markers with mononucleotide repeats (BAT-25, BAT-26, NR-21, NR-24, and NR-27), as previously described^28^. The EBV status was determined at Samsung Medical Center by EBV-encoded small RNA (EBER) in situ hybridization using standard protocols^29^.

### Statistical analysis

Statistical analysis was performed using IBM SPSS Statistics for Windows version 25.0 (IBM, Chicago, IL, USA) and the R-4.0.2 program for Mac OS. The chi-squared and Fisher’s exact tests were used to correlate the histopathologic features of pathological tissues and responsiveness using SPSS statistics. Heatmap representation of the exploration and validation cohorts was performed according to the study by Stein et al.^15^. Logistic regression analysis was used to compare histological features and IHC expression sites. For logistic regression and odds ratio analyses, the *glm()* function of *stat*, as well as the backward elimination function of *step()* (both in R package) was used. Survival analyses were performed utilizing the *survival* R package. Kaplan–Meier survival curves were used to estimate the patterns of progression-free survival (PFS) and overall survival (OS). For Kaplan–Meier curve survival analysis, a log-rank test (*surv_pvalue()* function) was used to compare survival curves. In the analysis of differentially expressed genes (DEGs), the fold change value was set to be less than 2, and the *P* value was set to be greater than 0.05. The collective 2-sided binomial Mann–Whitney–Wilcoxon test was used to compare differences between 2 groups. Linear correlations were determined using the Pearson correlation method. To classify the expression of each gene into a binary category, the *ROC*() function of *Epi* in the R package was used. *P* values less than 0.05 were considered significant.

### Predictive model generation and cross-validation

The *carat* R package was used in the model to predict responders versus nonresponders. We used the C5.0 decision tree model^30^ and random forest for classification^31^. To estimate the ‘out-of-bag’ area under the curve (AUC), a 10-fold cross-validation was repeated 500 times, and the best model was extracted. The *winnow* parameter was set to “TRUE”; therefore, we tried to prevent overfitting by measuring whether the field was useful in advance for the input field and then excluding it if it was not useful and modeling it. For random forest training, we used parameters that generated an ensemble of 500 trees. Repeated 10-fold CV was used to most accurately compare model performance. To define the optimal number of variables, a grid with mtry parameters of 1, 2, 3, 4, 8, and 16 was created. The *roc*() function of the R package *proc* was used to compare the performance of each model.

## Results

### Patient characteristics

All patients were Korean. The median age of the patients was 58 y (range, 28–79 y), and the majority were men (57.8%) (Table 1). Poorly differentiated tumors comprised 64.7% of cases. Sixty-three (61.8%) patients were administered pembrolizumab, and 30 (29.4%) were administered nivolumab. The percentage of patients with positive expression of PD-L1, defined by CPS PD-L1 IHC ≥ 1, was 42.2%. Paired clinical data and RNA-seq data were available for 45 cases. The overall response was determined in 22 cases (21.5%).

**Table 1:** Baseline characteristics of the study population.

	Total (*N* = 102)
Age	
Median (range)	58.0 (28–79)
Sex	
Male	59 (57.8%)
Female	34 (33.3%)
Not available	9 (8.8%)
Histologic grade	
Well to moderately differentiated	26 (25.5%)
Poorly differentiated	66 (64.7%)
ICI treatment regimen	
Pembrolizumab	63 (61.8%)
Nivolumab	30 (29.4%)
Not available	9 (8.8%)
PD-L1 staining (DAKO 22C3)	
CPS ≥ 1%	43 (42.2%)
CPS < 1%	51 (50.0%)
Not available	8 (7.8%)
Data available for RNA-seq	45 (44.1%)
Data available for pathology	93 (91.2%)
Overall response	
CR	3 (2.9%)
PR	19 (18.6%)
SD	29 (28.4%)
PD	51 (50.0%)
Progression-free survival (months)	
Median (range)	3.07 (0.47–33.03)
Overall survival (months)	
Median (range)	6.72 (0.47–33.03)

*ICI* immune checkpoint inhibitors, *RNA-seq* RNA sequencing, *CPS* combined positive score, *CR* complete response, *PR* partial response, *SD* stable disease, *PD* progressive disease.

### Presence of signet ring cells in nonresponders to immune checkpoint inhibitors

**Fig. 1:**
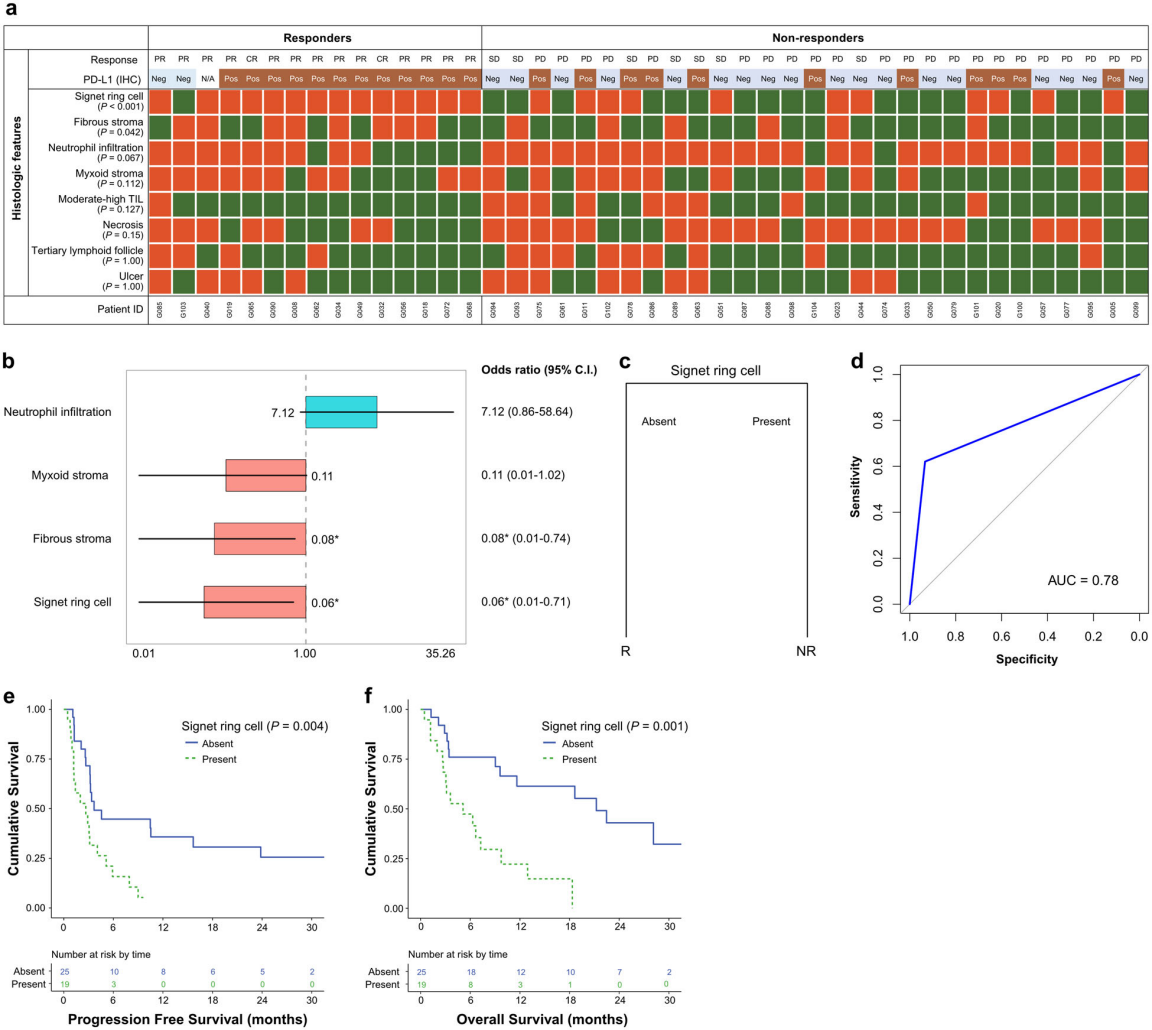
Predictive analysis of the responsiveness to ICIs on histopathological examinations of surgically excised specimens. **a** Heatmaps of individual histologic features in surgically excised specimens. Green indicates that the feature is present. Red indicates that the feature is absent. **b** Forest plot of the odds ratios of histological features. **c** ICI treatment responsiveness trained prediction model with histological features. **d** Receiver operating characteristic (ROC) curve showing the estimated AUC of the performance of the trained model. **e** Progression-free survival using the presence of SRC. **f** Overall survival using the presence of SRC. PR, partial response; CR, complete response; PD, progressive disease; SD, stable disease; R, responder; NR, nonresponder; ICI, immune checkpoint inhibitor; SRC, signet ring cell; CPS, combined positive score; Pos, positive (CPS ≥ 1); Neg, negative (CPS < 1); AUC, area under the curve; N/A, not available. *P < 0.05, as determined by multivariate logistic regression analysis.

We examined the differences in the histological features between the responder and nonresponder groups using the surgically resected specimens. Histopathological examination revealed that presence of SRC or fibrous stroma were more correlated with the nonresponders than the responders (*P* < 0.001 and *P* = 0.042, respectively) (Fig. 1a). We observed only a single case of SRC among responders. Using the backward elimination of variables in the logistic regression analysis, we obtained an odds ratio for SRC of 0.06 (95% confidence interval, CI: 0.01–0.71; *P* < 0.05) (Fig. 1b). Similarly, the odds ratio for fibrous stroma was found to be 0.08 (95% CI: 0.01–0.74; *P* < 0.05) (Fig. 1b). We accordingly trained our predictive model using only histological features by employing the *caret* R package. We used the C5.0 decision tree for classification and winnow to prevent overfitting. We repeated the 10-fold cross-validation 500 times, and the best model was identified to be a single variable model with or without SRC, with an AUC value of 0.78 (Fig. 1c, d). Survival analysis showed significantly longer progression-free survival (PFS) (log-rank *P* = 0.004) and overall survival (OS) (log-rank *P* = 0.001) in patients without SRC than in those with SRC (Fig. 1e, f). To validate its applicability in clinical practice, we applied the trained model to biopsy tissues.

However, we found that in biopsy tissues, SRC did not show a significant difference between responders and nonresponders (*P* = 0.283) (Fig. S2a). In contrast, there was a significant difference observed in TILs (*P* = 0.041) (Fig. S2a). It should be mentioned, however, that as there were only four specimens of responders among the examined biopsy tissues, there was a possibility of statistical limitation. Moreover, no SRC was detected, whereas TILs were present in all four responder specimens. We also performed survival rate analysis in these biopsy tissues and found that SRC was not significantly different regarding PFS (log-rank *P* = 0.641) or OS (log-rank *P* = 0.216) (Fig. S2b, c). However, the grade of TILs was demonstrated to be significant for both PFS (log-rank *P* = 0.007) and OS (log-rank *P* = 0.001) (Fig. S2d, e). Following the application of the trained model to biopsy tissues, we evaluated its accuracy to be 46.8% (22/47) (Fig. S2f).

### Gene set enrichment analysis of differentially expressed genes for identifying immune-related gene sets

We performed GSEA to explore the signaling pathways related to the DEGs and evaluate their biological significance (Fig. S3a). We observed that the interferon-γ response set (enrichment score [ES] = 0.66, normalized enrichment score [NES] = 1.60, nominal *P* = 0.079 and FDR *q*-value = 1.0) and interferon-α response set (ES = 0.70, NES = 1.55, nominal *P* = 0.097 and FDR q-value = 0.884) were enriched in responders (Fig. S3b), although not significant at FDR < 0.25. This finding suggested that responders and nonresponders could have different immune-related bioactivities. To explore individual patients, we analyzed the differences in gene set enrichment using the “ssgsea” module. Accordingly, the obtained dendrograms showed that several signaling pathways, such as the MYC target, TGF-β signaling, interferon-α response, interferon-γ response, TNF-α signaling via NF-κb, IL2-STAT5 signaling, IL6-JAK-STAT3 signaling, and inflammatory response, differed between responders and nonresponders (Fig. S3c). This result suggested the possibility of the presence of immunologically distinct differences between responders and nonresponders.

### The *CXCL11* gene was highly expressed in responders to immune checkpoint inhibitors

**Fig. 2:**
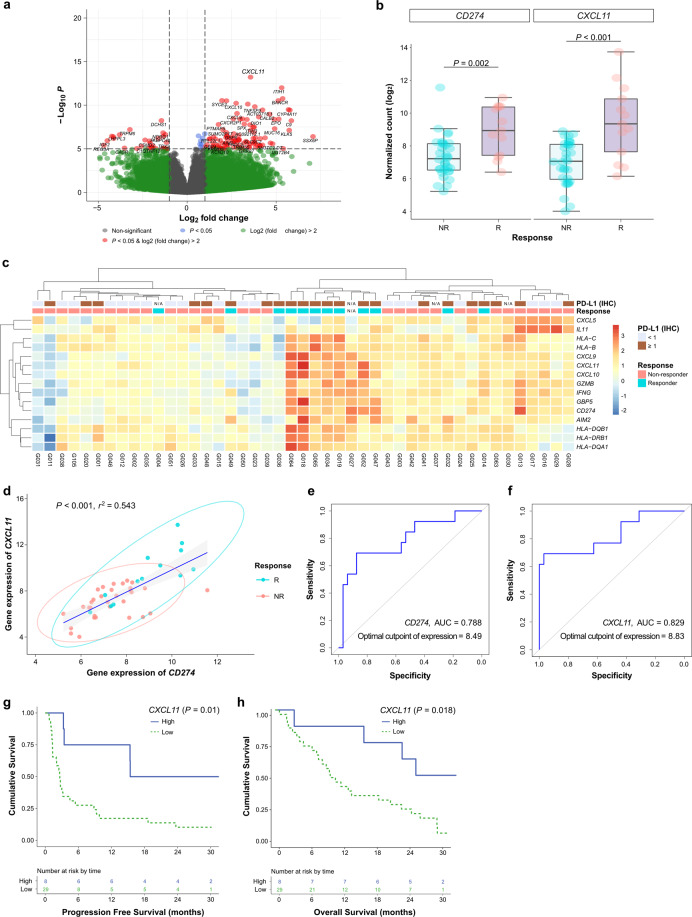
Analysis of differentially expressed genes (DEGs) in RNA sequencing data related to the ICI treatment response. **a** Volcano plot showing DEGs based on the response to ICIs with log2 (fold change in expression) >2 and *P* < 0.05. **b** Boxplot of gene expression values (DESEQ2) of *CD274* and *CXCL11* based on the ICI response. **c** Heatmap of representatively significant immune-related genes in the analysis of DEGs. **d** Correlation of the gene expression of *CXCL11* and *CD274*. **e** Predictive value and optimal cutoff value of the *CD274* gene for responsiveness using the ROC curve. **f** Predictive value and optimal cutoff value of the *CXCL11* gene using the ROC curve. **g** Progression-free survival using the split level by the optimal cutoff point of *CXCL11* for responsiveness using the ROC curve. **h** Overall survival using the split level by the optimal cutoff value of *CXCL11* for responsiveness using the ROC curve. R, responder; NR, nonresponder; IHC, immunohistochemistry; ICI, immune checkpoint inhibitor; ROC, receiver operating characteristic; N/A, not available.

We conducted DEG analysis on our RNA-seq data using DESEQ2 to find a predictable biomarker that would be superior to or support the PD-L1 (IHC) test. We found that *CXCL11* showed a log 2-fold change of 3.56 greater in responders than in nonresponders (adjusted *P* < 0.001) (Fig. 2a). Interestingly, *CXCL11* showed a greater difference (*P* < 0.001) than *CD274* (*P* = 0.002), the gene encoding the PD-L1 protein (Fig. 2b). In addition, our generated heatmaps showed higher expression of immune-related genes, especially *CXCL11*, in responders than in nonresponders (Fig. 2c). We further found that expression of the *CXCL11* gene was correlated with that of *CD274* (*P* < 0.001, *r*^2^ = 0.543) (Fig. 2d). Receiver operating characteristic (ROC) curve analysis of responsiveness was based on the expression of the *CD274* and *CXCL11* genes. The optimal cutoff point for the expression of *CD274* was 8.49, with an AUC of 0.788 (Fig. 2e). Likewise, the optimal cutoff point for the expression of *CXCL11* was 8.83, with an AUC of 0.829 (Fig. 2f). To use the expression of a single gene as a biomarker based only on the RNA-seq data, we considered the expression of *CXCL11*. To determine whether *CXCL11* was correlated with prognosis, we performed survival analysis using the optimal cutoff values for gene expression. The expression level of *CXCL11* was shown to be significant in both PFS (log-rank *P* = 0.01) and OS (log-rank *P* = 0.018) (Fig. 2g, h).

### CXCL11 was highly expressed in the tumor cell cytoplasm of responders to immune checkpoint inhibitors

**Fig. 3:**
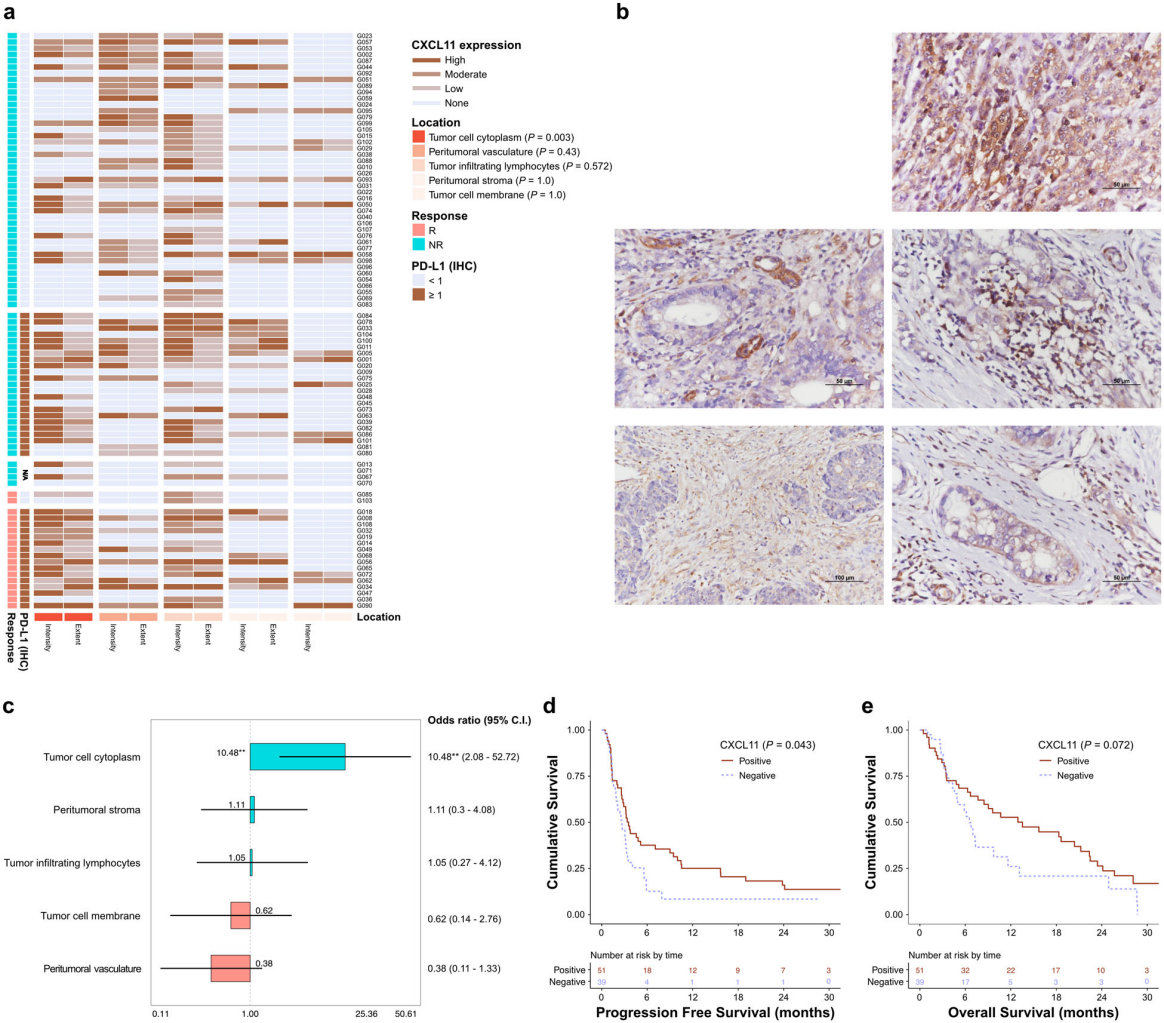
Evaluation of ICI treatment responsiveness based on immunohistochemical staining for the expression of CXCL11. **a** Heatmap with intensity and extent depending on the location of the expression of CXCL11. **b** Representative photomicrographs showing the expression of CXCL11 in gastric cancer tissue. Expression in the tumor cell cytoplasm (top; original magnification ×400), peritumoral vasculature (top, left; original magnification ×400), tumor-infiltrating lymphocytes (middle, right; original magnification ×400), peritumoral stroma (bottom, left; original magnification ×200), and tumor cell membrane (bottom, right; original magnification ×400). **c** Forest plot of the odds ratio of the expression location of CXCL11. **d** Progression-free survival using the expression of CXCL11. **e** Overall survival using the expression of CXCL11. R, responder; NR, nonresponder; ICI, immune checkpoint inhibitor; IHC, immunohistochemistry; CPS, combined positive score; N/A, not available. *P < 0.05 and **P < 0.01, as determined by multivariate logistic regression analysis.

Based on the findings revealing the significance of *CXCL11* at the transcript level, we performed IHC to reveal its protein expression in tissues. Accordingly, we evaluated the expression of CXCL11 in all stained cells and determined its location, intensity, and extent (Fig. 3a, b). CXCL11 was observed to be expressed mainly in the cytoplasm and locally in the cell membrane of tumor cells. In addition, we detected its local presence in interstitial tissues and blood vessels (venules or capillaries) around tumor nests. The expression of CXCL11 in the cytoplasm of tumor cells was demonstrated to be significantly correlated with the response to ICIs (*P* = 0.003) (Fig. 3a). Logistic regression analysis revealed that the expression of CXCL11 in the cytoplasm was the most significant (odds ratio = 10.48; 95% CI: 2.08–52.72; *P* < 0.01) (Fig. 3c). Therefore, we determined that the IHC results of CXCL11 were either positive or negative according to its expression in the cytoplasm of tumor cells. Similar to the *CXCL11* gene shown to be significantly associated with prognosis at the transcript level, the CXCL11 protein was found to be significant for PFS (log-rank *P* = 0.043) but not OS (log-rank *P* = 0.072) (Fig. 3d, e).

### Integrative predictive modeling for the response to immune checkpoint inhibitors

**Fig. 4:**
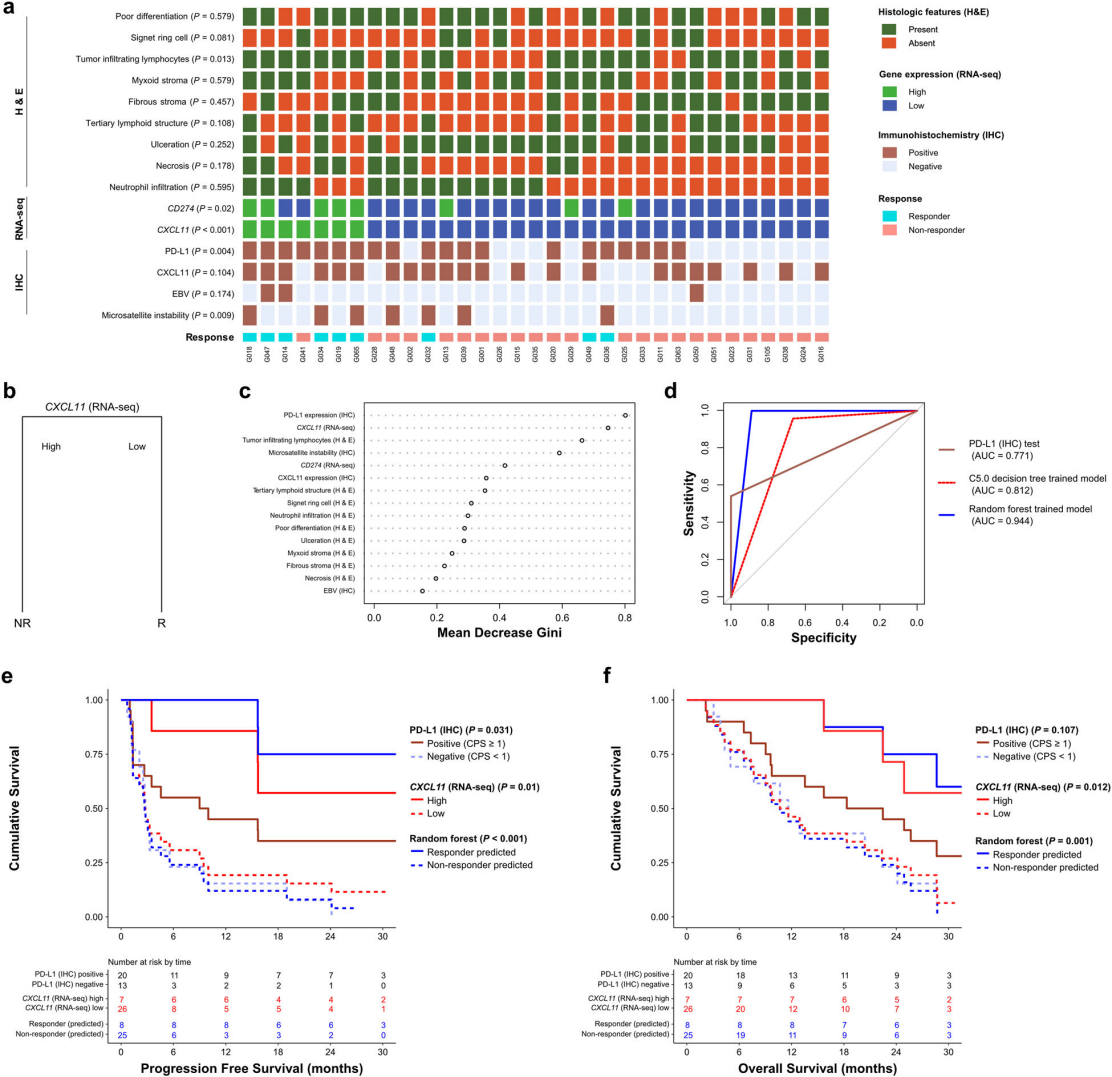
ICI responsiveness prediction model trained with integrated data and performance evaluation. **a** Heatmap showing the result of integrating all results from histological, RNA-seq, and IHC analyses to train the predictive model. Patients with missing data were removed. **b** The predictive model trained using the C5.0 decision tree model. The categorical level of the *CXCL11* (RNA-seq) single variable was trained as an optimal model. **c** The mean decrease in Gini of the predictive model trained using the random forest model. **d** Comparative ROC curve showing the AUC of the performance of the trained model in **b** and **c. e**, **f** Comparative analysis of the survival rate between the predicted responsiveness using the trained models and the PD-L1 (IHC) test. **e** Progression-free survival. **f** Overall survival. R, responder; NR, nonresponder; ICI, immune checkpoint inhibitor; H/E, hematoxylin and eosin; IHC, immunohistochemistry; ROC, receiver operating characteristic; AUC, area under the curve; RNA-seq, RNA sequencing.

As mentioned above, we attempted to predict the individual histological, transcriptional, and immunohistochemical responses. We thus set out to develop predictive models integrating histopathologic, transcriptomic, and immunohistochemical data to predict responders versus nonresponders. There were 33 patients in our cohort for whom all data, including histopathology, RNA-seq, and IHC results, were available (Fig. 4a). Using Fisher’s exact test in this data set, we found that TILs (H&E; *P* = 0.013), *CD274* (RNA-seq; *P* = 0.02), *CXCL11* (RNA-seq; *P* < 0.001), PD-L1 (IHC; *P* = 0.004), and MSI (IHC, *P* = 0.009) were significantly associated with responsiveness (Fig. 4a). We hence trained our predictive model using the *caret* R package and the C5.0 decision tree method for classification and random forest classification. For both the random forest and C5.0 decision trees, we performed 10-fold cross-validation 500 times, and for the C5.0 decision tree, we performed winnowing to prevent overfitting. When trained with the C5.0 decision model, the categorical level of the expression of *CXCL11*, a single variable, was demonstrated to be the best model (Fig. 4b). The AUC of the C5.0-trained model was 0.812 (Fig. 4d). When trained with the random forest model, the out-of-bag (OOB) estimate of the error rate of the best model was shown to be 22.73%. We observed that PD-L1 (IHC), *CXCL11* (RNA-seq), TILs (H&E), and MSI (IHC) were important variables in the random forest-trained model (Fig. 4c). The AUC of the random forest-trained model was 0.944 (Fig. 4d). Interestingly, both models showed better prediction performance than the PD-L1 (IHC) test (AUC = 0.771) (Fig. 4d). We compared the survival outcomes with the predicted results of the trained models and those of the PD-L1 (IHC) test. Comparison of the survival results using the *CXCL11* (RNA-seq) categorical variable revealed a more significant difference for both PFS (log-rank *P* = 0.01) and OS (log-rank *P* = 0.012) between responders and nonresponders in the model trained with the C5.0 decision tree compared with the results predicted with the PD-L1 (IHC) test (log-rank *P* = 0.031 for PFS; log-rank *P* = 0.107 for OS) (Fig. 4e, f). Survival analysis using the random forest-trained model, which showed better performance than the C5.0 decision tree-trained model, exhibited the most significant difference between responders and nonresponders (log-rank *P* < 0.001 for PFS; log-rank *P* = 0.001 for OS) (Fig. 4e, f).

## Discussion

In this study, we analyzed a clinically annotated cohort of patients with advanced gastric cancer treated with anti-PD-L1 ICIs with available histopathology, RNA-seq, and IHC data. We analyzed each datum individually, as well as integrally. In their study, Kim et al showed that MSI-H and EBV (+) were reliable biomarkers for immunotherapy, as well as for PD-L1 IHC^4^. They suggested that patients with EBV (+) gastric cancer be actively considered for pembrolizumab monotherapy, similar to those with MSI-H tumors. However, research on histopathological biomarkers other than genetic variations has been insufficient. In many cases, patients with gastric cancer are diagnosed through endoscopic biopsy. As the biopsy tissue is markedly smaller than the surgically excised tissue, a single PD-L1 IHC test alone would not provide a sufficiently accurate result to predict the efficacy of a drug. Thus, the prediction of nonresponders based only on histological features prior to the PD-L1 IHC test might prevent unnecessary testing and treatment. In particular, we sought to identify histological features that pathologists could easily identify in small biopsy tissues. For instance, the presence of SRC is considered a representative histological finding in gastric cancer with poor differentiation and has been linked to a poor prognosis^32^. In this study, the presence of SRC was shown to be important in predicting the efficacy of ICIs. However, there have been few reports on the clinical impact of ICI markers on SRC in gastric cancer. Jin et al. reported the expression of PD-1, PD-L1, T cell infiltration, MSI, and EBV, as well as the relationship of each factor with survival, in 89 patients with advanced SRC carcinoma^33^. Although the patients in that study were not treated with ICIs, it was the only investigation reporting an association of SRC with PD-L1. PD-1, PD-L1, T cell infiltration, MSI, and EBV did not affect prognosis. In other words, the presence of SRC, which does not appear to be influenced by other factors, might be an independent variable in predicting prognosis. Therefore, the presence of SRC in nonresponders to ICIs (i.e., PD-L1) might be an important finding in our study. Importantly, our study provides the first evidence of a high nonresponse rate to pembrolizumab in SRC tumors. The presence of SRC and TILs in small biopsy tissues at the time of diagnosis by a pathologist could be used to predict responsiveness to ICIs. This result could be helpful even in a clinical environment where the latest molecular pathology diagnostic equipment might not be available.

In the tumor microenvironment, the *CXCL9*/*10*/*11*-*CXCR3* signaling pathway is known to primarily promote the chemotactic movement of *CXCR3*-activated immune cells into the tumor site for antitumor immunity^34^. Zhang et al. reported that *CXCL11-CXCR3* upregulated the expression of PD-L1 by activating the *STAT* and *PI3K-Akt* signaling pathways in gastric cancer cells in vitro^35^. Genetic analysis of DEGs using RNA sequencing data has suggested the possibility of molecular markers and their usefulness in IHC settings. In this study, DEG analysis using RNA sequencing data revealed that the expression of *CXCL11*, *CXCL9*, and *CXCL10* was significantly high in responders to ICIs (Fig. 2a, c). Among them, *CXCL11*, which showed the highest accuracy, was found to be positively correlated with the expression of the *CD274* gene (*r*^*2*^ = 0.543) (Fig. 2d), consistent with the results reported by Zhang et al.^35^.

Univariate analysis revealed that IHC staining of CXCL11 was associated with the responsiveness to ICIs. However, this was not shown to be significant in the multivariate analysis. This discrepancy could be attributed to several factors. First, there was a low correlation between the expression level of CXCL11 in IHC and its expression level in RNA sequencing. The correlation between the expression of the *CD274* gene and the PD-L1 (IHC) test was low (*r*^*2*^ = 0.202), as was the correlation between the expression of the *CXCL11* gene and the IHC staining of CXCL11 (*r*^*2*^ = 0.107) (Fig. S4a, b). This difference might have occurred because the bulk tissue for RNA sequencing included various cells, such as inflammatory, stromal, blood vessel, and tumor cells. Second, *CXCL11* and PD-L1 are not independent variables, as they were previously shown to be affected by each other^35^. The IHC results of CXCL11 were not included in the final model predicting the effect of PD-L1 IHC in our multivariate analysis. Further research is needed to identify immunohistochemical markers that could overcome the limitations of the PD-L1 IHC test.

Research using RNA-seq data allows the analysis of not only differentially expressed genes, as in this study, but also of alternative splicing, variants, structural variants, and neoantigens. As described above, while analyzing DEGs using the bcbio-nextgen bioinformatics framework (version 1.2.4)^18^, we simultaneously performed analyses of alternative splicing, genetic variation, and structural variation. Briefly, after the sequencing results were arranged in STAR^19^, genetic variant calling was performed using the gatk-3.8 HaplotypeCaller^36^ followed by VEP^37^, while alternative splicing analysis was performed using rmats-4.1.0^38^, and structural variation analysis was performed using arriba^39^. However, each analysis tool needed to be further validated so that the expected results would be consistent with the actual results, and thus, it was difficult to use these results in the current study. Further research on the results of each analysis tool is needed.

This study trained data using a decision model to determine the best model for classification among variables. The random forest is widely used as the most popular method to show strong predictive power. This model also showed far superior predictive performance than the C5.0 decision tree model. The random forest method has been shown to demonstrate excellent predictive power to the trained machine, but it is difficult to intuitively interpret the model for use by clinicians in a clinical setting due to the black box problem. In this random forest model, the ROC curve was used to select the optimal model using the largest value. The final value used for the model was mtry = 1. *CXCL11* (RNA-seq) was the model with the best performance even when all decision trees with one variable were randomly extracted from the random forest. The model trained with C5.0 was also a decision tree with one *CXCL11* (RNA-seq) variable. Although its performance was demonstrated to be inferior to that of the random forest model, simple decision-making models could be more helpful to clinicians in the actual clinical setting. This study did not intend to compare the performance of machine learning methods. Prediction based on the presence or absence of SRC when only histopathological information is available or based on the level of *CXCL11* in the presence of RNA-seq information could be simpler and more helpful in the actual clinical setting.

One of the difficulties in integrating each data point to train a new biomarker model was the missing data for each case. In this study, 100 cases were subjected to a histopathological evaluation, and 45 cases had RNA-seq data, but only 33 cases had both. We attempted to use the whole exome sequencing data of patients from a previous study conducted by the research team of Professor Lee^4^ who overlapped with those in this study. Since there are well-known biomarkers, in whole exome sequencing data, such as tumor mutational burden and MSI, we also tried to use exome sequencing data for comparative analysis with the biomarkers found in this study. However, due to missing data in each case, it was difficult to use a full data set to train the model. To integrate each biomarker and compare their predictive performance, further studies of models trained with a larger number of data sets without missing data are required.

## Supplementary information

Supplemental Figures

Supplemental Tables

## Data Availability

All data have been deposited into the European Nucleotide Archive and are available under accession code PRJEB25780.

## References

[1] Taube JM (2018). Implications of the tumor immune microenvironment for staging and therapeutics. Mod. Pathol..

[2] Samstein RM (2019). Tumor mutational load predicts survival after immunotherapy across multiple cancer types. Nat. Genet..

[3] Janjigian YY (2018). Genetic predictors of response to systemic therapy in esophagogastric cancer. Cancer Discov..

[4] Kim ST (2018). Comprehensive molecular characterization of clinical responses to PD-1 inhibition in metastatic gastric cancer. Nat. Med..

[5] Wang, F. et al. Safety, efficacy and tumor mutational burden as a biomarker of overall survival benefit in chemo-refractory gastric cancer treated with toripalimab, a PD1 antibody in phase Ib/II clinical trial NCT02915432. *Ann. Oncol*. **30**, 1479–1486 (2019). 10.1093/annonc/mdz197 PMC6771223 31236579

[6] Kim JW (2016). Prognostic implications of immunosuppressive protein expression in tumors as well as immune cell infiltration within the tumor microenvironment in gastric cancer. Gastric Cancer.

[7] Lee H (2008). Prognostic implications of type and density of tumour-infiltrating lymphocytes in gastric cancer. Br. J. Cancer.

[8] Kim, J. Y., Kim, W. G. & Kwon, C. H. Differences in immune contextures among different molecular subtypes of gastric cancer and their prognostic impact. *Gastric Cancer*, **22**, 1–12 (2019). 10.1007/s10120-019-00974-4 31152268

[9] Kwak, Y., Seo, A. N., Lee, H. E. & Lee, H. S. Tumor immune response and immunotherapy in gastric cancer. *Korean J. Pathol.* **54**, 20 (2019). 10.4132/jptm.2019.10.08 PMC6986974 31674166

[10] Kulangara K (2018). Clinical utility of the combined positive score for programmed death ligand-1 expression and the approval of pembrolizumab for treatment of gastric cancer. Arch. Pathol. Lab. Med..

[11] Fuchs CS (2018). Safety and efficacy of pembrolizumab monotherapy in patients with previously treated advanced gastric and gastroesophageal junction cancer: phase 2 clinical KEYNOTE-059 trial. JAMA Oncol..

[12] Kang Y-K (2017). Nivolumab in patients with advanced gastric or gastro-oesophageal junction cancer refractory to, or intolerant of, at least two previous chemotherapy regimens (ONO-4538-12, ATTRACTION-2): a randomised, double-blind, placebo-controlled, phase 3 trial. Lancet.

[13] Muro K (2016). Pembrolizumab for patients with PD-L1-positive advanced gastric cancer (KEYNOTE-012): a multicentre, open-label, phase 1b trial. Lancet Oncol..

[14] Cottrell T (2018). Pathologic features of response to neoadjuvant anti-PD-1 in resected non-small-cell lung carcinoma: a proposal for quantitative immune-related pathologic response criteria (irPRC). Ann. Oncol..

[15] Stein J (2019). Major pathologic response on biopsy (MPRbx) in patients with advanced melanoma treated with anti-PD-1: evidence for an early, on-therapy biomarker of response. Ann. Oncol..

[16] Eisenhauer EA (2009). New response evaluation criteria in solid tumours: revised RECIST guideline (version 1.1). Eur. J. Cancer.

[17] Hendry S (2017). Assessing tumor infiltrating lymphocytes in solid tumors: a practical review for pathologists and proposal for a standardized method from the International Immuno-Oncology Biomarkers Working Group: Part 1: Assessing the host immune response, TILs in invasive breast carcinoma and ductal carcinoma in situ, metastatic tumor deposits and areas for further research. Adv. Anat. Pathol..

[18] Brad C. et al. bcbio/bcbio-nextgen: v1.2.4. (2020). 10.5281/zenodo.4041990

[19] Dobin A (2013). STAR: ultrafast universal RNA-seq aligner. Bioinformatics.

[20] Li H (2009). The sequence alignment/map format and SAMtools. Bioinformatics.

[21] Ewels P, Magnusson M, Lundin S, Käller M (2016). MultiQC: summarize analysis results for multiple tools and samples in a single report. Bioinformatics.

[22] Zerbino DR (2017). Ensembl 2018. Nucleic Acids Res..

[23] Liao Y, Smyth GK, Shi W (2013). featureCounts: an efficient general purpose program for assigning sequence reads to genomic features. Bioinformatics.

[24] Love MI, Huber W, Anders S (2014). Moderated estimation of fold change and dispersion for RNA-seq data with DESeq2. Genome Biol..

[25] Subramanian A (2005). Gene set enrichment analysis: a knowledge-based approach for interpreting genome-wide expression profiles. Proc. Natl Acad. Sci. USA.

[26] Mootha VK (2003). PGC-1α-responsive genes involved in oxidative phosphorylation are coordinately downregulated in human diabetes. Nat. Genet..

[27] Hänzelmann S, Castelo R, Guinney J (2013). GSVA: gene set variation analysis for microarray and RNA-seq data. BMC Bioinformatics.

[28] Seiwert TY (2016). Safety and clinical activity of pembrolizumab for treatment of recurrent or metastatic squamous cell carcinoma of the head and neck (KEYNOTE-012): an open-label, multicentre, phase 1b trial. Lancet Oncol..

[29] Song HJ (2010). Host inflammatory response predicts survival of patients with Epstein-Barr virus–associated gastric carcinoma. Gastroenterology.

[30] Bujlow, T., Riaz, T. & Pedersen, J. M. In 2012 international conference on computing, networking and communications (ICNC). 237–241 (IEEE).

[31] Svetnik V (2003). Random forest: a classification and regression tool for compound classification and QSAR modeling. J. Chem. Inf. Comput. Sci..

[32] Nagtegaal, I. D. et al. The 2019 WHO classification of tumours of the digestive system. *Histopathology*. **76**, 182–188 (2019). 10.1111/his.13975 PMC7003895 31433515

[33] Jin S (2017). The PD-1, PD-L1 expression and CD3+ T cell infiltration in relation to outcome in advanced gastric signet-ring cell carcinoma, representing a potential biomarker for immunotherapy. Oncotarget.

[34] Shahabuddin S (2006). CXCR3 chemokine receptor-induced chemotaxis in human airway epithelial cells: role of p38 MAPK and PI3K signaling pathways. Am. J. Physiol. Cell Physiol..

[35] Zhang C (2018). CXCL9/10/11, a regulator of PD-L1 expression in gastric cancer. BMC Cancer.

[36] Poplin, R. et al. Scaling accurate genetic variant discovery to tens of thousands of samples. Preprint at https://www.biorxiv.org/content/10.1101/201178v3 (2017).

[37] McLaren W (2016). The ensembl variant effect predictor. Genome Biol..

[38] Shen S (2014). rMATS: robust and flexible detection of differential alternative splicing from replicate RNA-Seq data. Proc. Natl Acad. Sci. USA.

[39] Uhrig, S. Arriba - Fast and accurate gene fusion detection from RNA-Seq data https://github.com/suhrig/arriba/ (2019).

