# Supplementary material for: Practical prediction model of the clinical response to programmed death-ligand 1 inhibitors in advanced gastric cancer
Source: Exp Mol Med. 2021 Feb 5;53(2):223–34. doi: 10.1038/s12276-021-00559-1 (PMC8080676; doi:10.1038/s12276-021-00559-1)
Supplement: Supplementary file 1 — Supplemental Figures [file 12276_2021_559_MOESM1_ESM.pdf]

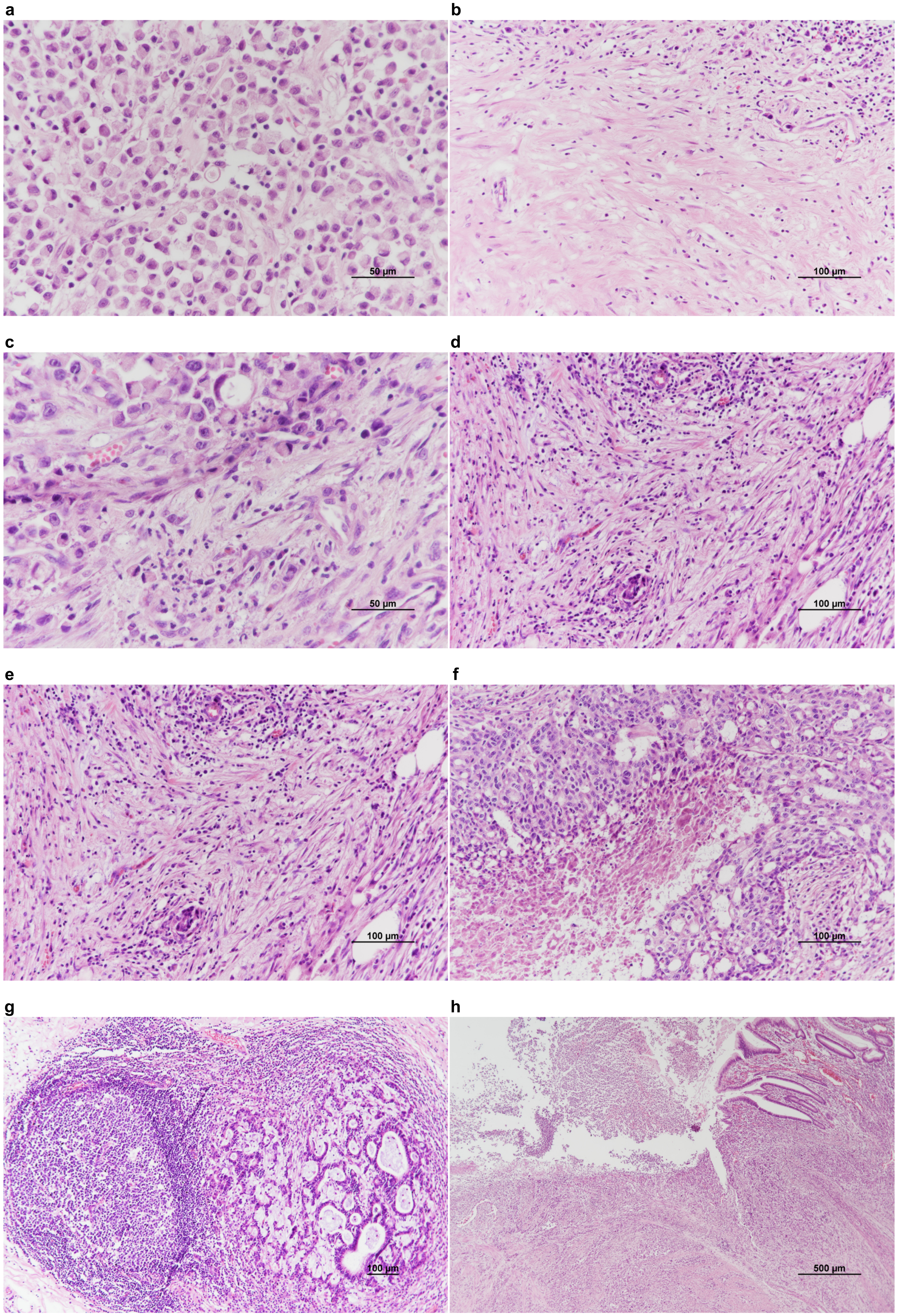

Fig. S1. Representative photographs of the histopathologic features used in this study. **a,** Signet ring cell, original magnification ×400. **b,** Fibrous stroma, original magnification ×200. **c,** Neutrophil infiltration, original magnification ×400. **d,** Myxoid stroma, original magnification ×200. **e,** Tumor-infiltrating lymphocytes, original magnification ×200. **f,** Tumor-necrosis, original magnification ×200. **g,** Tertiary lymphoid structure, original magnification ×200. **h,** Ulceration, original magnification ×40. All panels: hematoxylin and eosin staining.

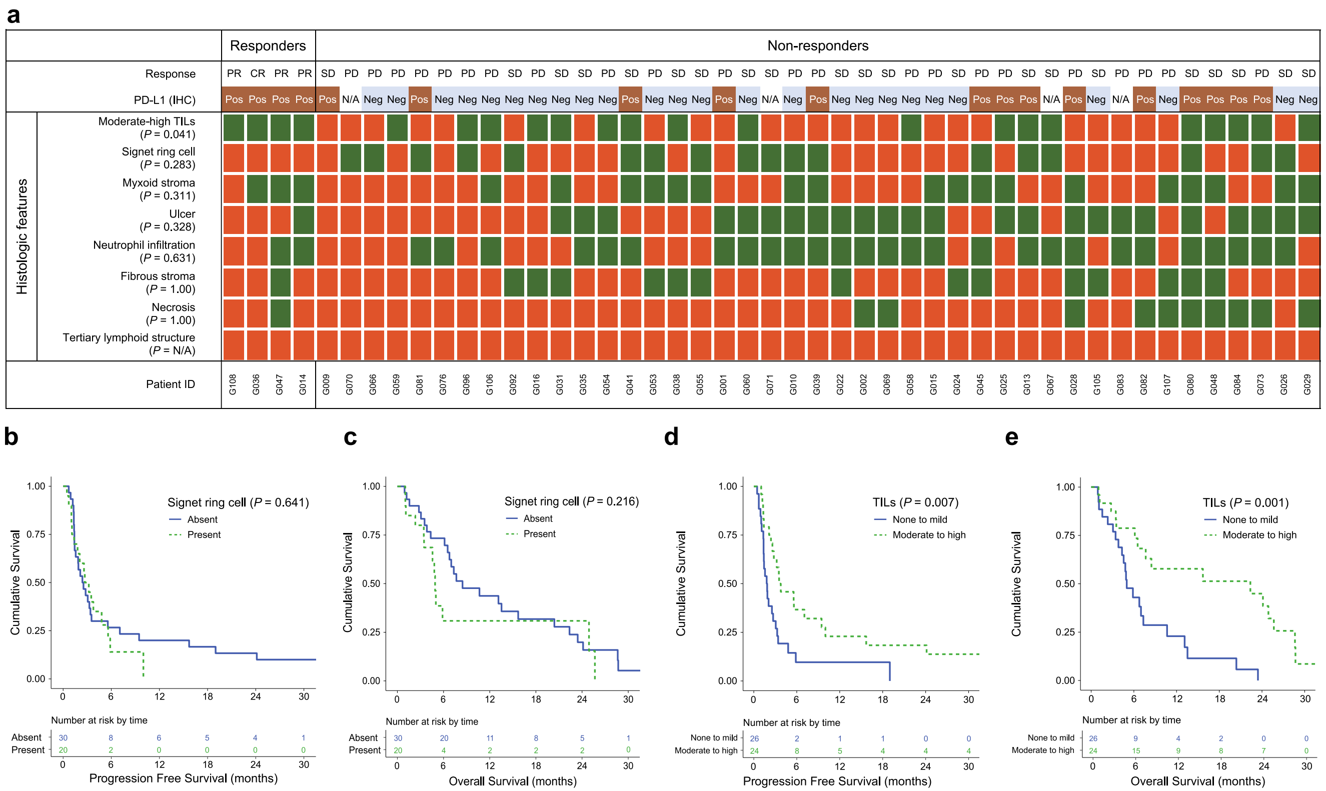

f

Biopsied specimen (N = 47)	Predicted (tested)	
Actual	Responder	Non-responder
Responder	4	0
Non-responder	25	18

Fig. S2 Predictive analysis of ICI responsiveness in histopathological examination of small biopsied specimens. **a,** Heatmaps of individual histologic features in small biopsied specimens. Green indicates that the feature is present. Red indicates that the feature is absent. **b,** Progression-free survival using the presence of SRC. **c,** Overall survival using the presence of SRC. **d,** Progression-free survival using the presence of TILs. **e,** Overall survival using the presence of TILs. **f,** Performance table applied to a small biopsy tissue using a model trained with histological features of a surgical specimen. PR, partial response; CR, complete response; PD, progressive disease; SD, stable disease; R, responder; NR, non-responder; ICI, immune checkpoint inhibitor; SRC, signet ring cell; R, partial response; CR, complete response; PD, progressive disease; SD, stable disease; CPS, combined positive score; Pos, positive (CPS ≥ 1); Neg, negative (CPS < 1); N/A, not available.

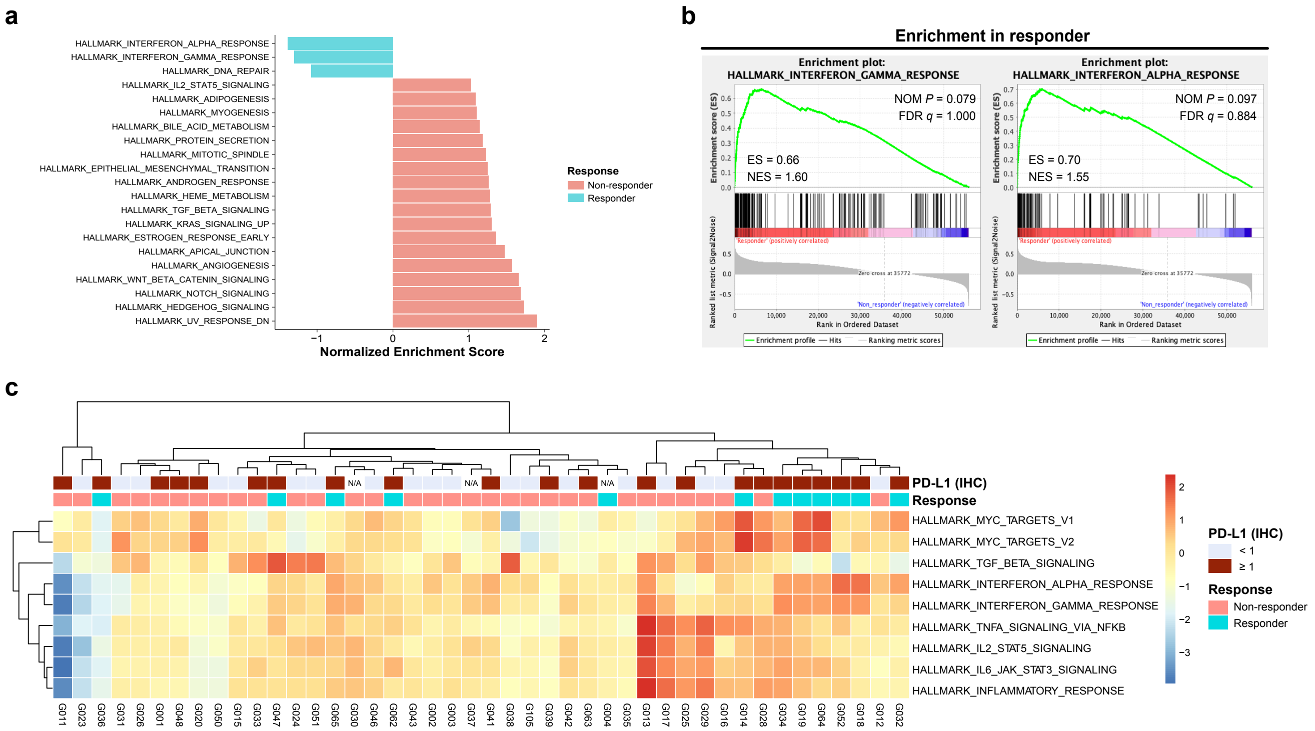

Fig S3. Gene set analysis. a, GSEA showed top 18 gene sets up-regulated in non-responders and top 3 gene sets up-regulated in responders. **b,** Top two gene sets of responders in the gene enrichment analysis. In responder group, the interferon alpha & gamma gene set (associated with immune system) was up-regulated to the top, but not statistically significant. (NOM $P = 0.079$ for interferon gamma response ; NOM $P = 0.097$ for interferon alpha response) **c,** Gene set variation analysis.

ES, enrichment score; NES, normalized enrichment score; NOM *P*, nominal *P*-value; IHC, immunohistochemistry

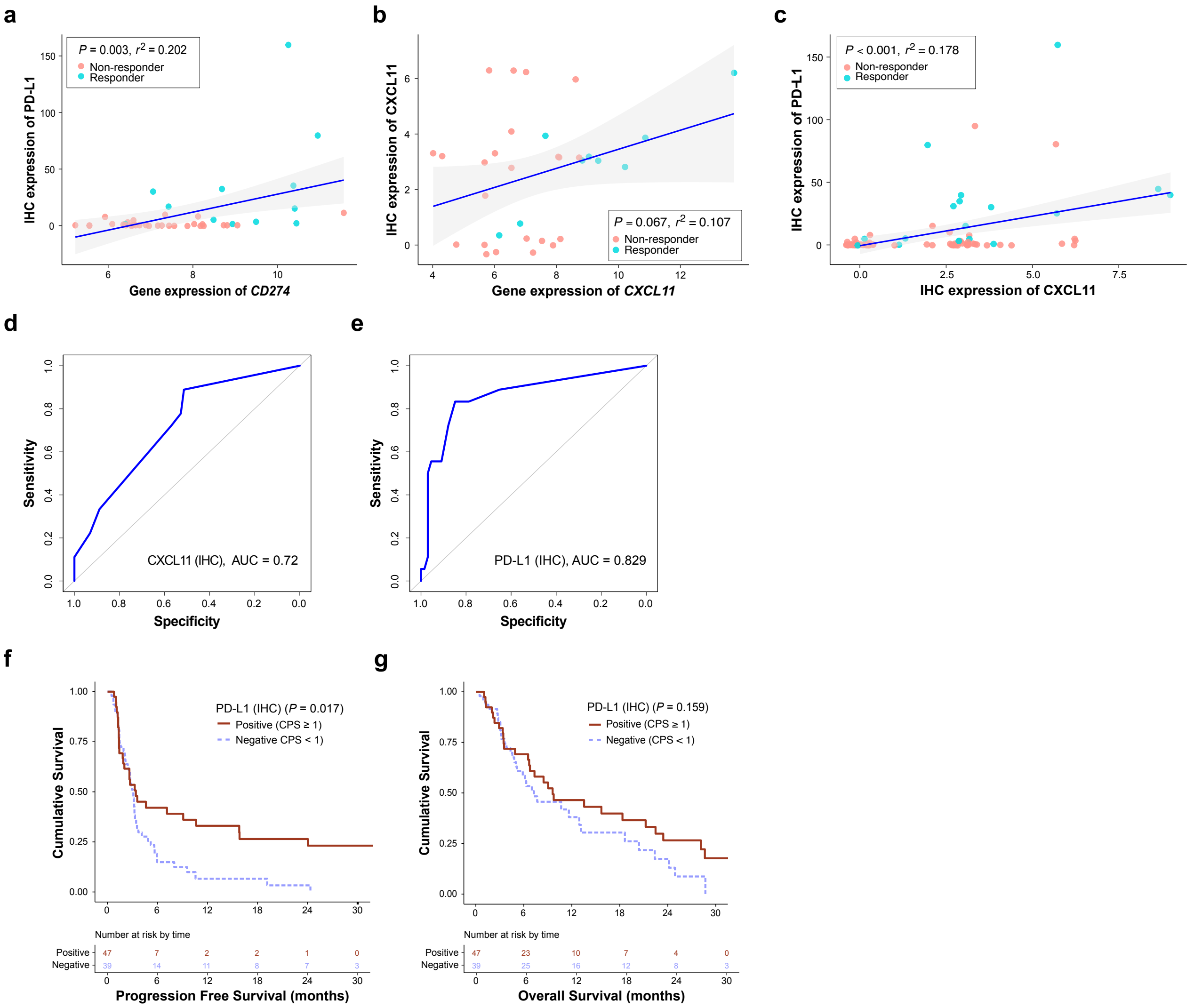

Fig S4. a, Correlation with the level of expression of the *CD274* gene in RNA seq and the level of protein expression of the PD-L1 gene in IHC. **b,** Correlation with the level of *CXCL11* gene expression in RNA-seq and the protein expression level of CXCL11 gene in IHC. **c,** Correlation with the level of expression of CXCL11 and the level of PD-L1 in IHC. **d,** Predictive value of CXCL11 (IHC) expression for responsiveness using ROC curve. **e,** Predictive value of PD-L1 (IHC) expression for responsiveness using ROC curve. **f-g** Survival analysis of the PD-L1 (IHC) test result (N = 86). **f,** Progression-free survival. **g,** Overall survival. IHC, immunohistochemistry; RNA-seq, RNA sequencing; ROC, receiver operating characteristic.
